# Association of obstructive sleep apnea plus hypertension and prevalent cardiovascular diseases: A cross-sectional study

**DOI:** 10.1097/MD.0000000000004691

**Published:** 2016-09-30

**Authors:** Ling Wang, Anping Cai, Jiawei Zhang, Qi Zhong, Rui Wang, Jiyan Chen, Yingling Zhou

**Affiliations:** Department of Cardiology, Guangdong Cardiovascular Institute, Guangdong Provincial Key Laboratory of Coronary Heart Disease Prevention, Guangdong General Hospital, Guangdong Academy of Medical Sciences, Guangzhou, China.

**Keywords:** cardiovascular diseases, hypertension, obstructive sleep apnea

## Abstract

Current study sought to evaluate the associations of obstructive sleep apnea (OSA) plus hypertension (HTN) and prevalent cardiovascular diseases (CVD).

This was a cross-sectional study and a total of 1889 subjects were enrolled. The apnea-hypopnea index (AHI) was measured by polysomnography and OSA degree was classified as mild (AHI 5–14.9) and moderate-severe (AHI ≥ 15), and AHI < 5 was considered no-OSA. Mean and lowest oxyhemoglobin saturation (SaO_2_) was detected by pulse oximetry. Between-group differences were assessed and logistic regression analysis was used to analyze the association of OSA plus HTN and prevalent CVD.

Compared to normotensive subjects, hypertensive subjects were older and had higher body mass index (BMI), neck girth, waist–hip ratio, AHI, and low-density lipoprotein cholesterol (LDL-C) level. Conversely, mean and lowest SaO_2_ levels were significantly lower. Logistic regression analysis showed that in an unadjusted model, compared to subjects with no-OSA and no-HTN (reference group), the association of HTN plus moderate-severe-OSA and prevalent CVD was the most prominent (odds ratio [OR]: 2.638 and 95% confidence interval [CI]: 1.942–3.583). In normotensive subjects, after adjusted for potential covariates, the associations of OSA (regardless of severity) and prevalent CVD were attenuated to nonsignificant. In hypertensive subjects, however, the associations remained significant but were reduced. Further adjusted for mean and lowest SaO_2_, the associations remained significant in HTN plus no-OSA (OR: 1.808, 95% CI: 1.207–2.707), HTN plus mild-OSA (OR: 2.003, 95% CI: 1.346–2.980), and HTN plus moderate-severe OSA (OR: 1.834, 95% CI: 1.214–2.770) groups.

OSA plus HTN is associated with prevalent CVD, and OSA may potentiate the adverse cardiovascular effects on hypertensives patients but not normotensives.

## 1 Introduction

Based on reports from epidemiological studies,^[1–4]^ the incidence and prevalence of obstructive sleep apnea (OSA) around the world are increased dramatically in recent decades. With respect to accumulating evidence demonstrating the adverse effects OSA exerts on cardiovascular system, OSA currently is considered as a major and independent risk factor of cardiovascular diseases (CVD).^[5]^ Mechanistically, high-frequency intermittent hypoxemia with associated reduced oxyhemoglobin saturation (SaO_2_) caused by repetitive upper airway obstruction leads to systemic inflammation, oxidative stress, endothelia dysfunction, and sympathetic nervous activation^[6–9]^ and all these pathological alterations are detrimental to cardiovascular system_._

In recent decades, a substantial amount of epidemiological studies have revealed that OSA is highly prevalent in patients with arterial hypertension (HTN),^[3,10]^ and OSA may render additional CVD risk in hypertensive subjects.^[11–13]^ Therefore, it is clinically relevant to screen and treat OSA effectively and efficiently so as to reduce the CVD risk associated with both HTN and OSA. With profound alteration of lifestyle, the prevalence of HTN and obesity, the 2 major risk factors of OSA,^[14,15]^ in China are considerably increased in recent 3 decades. Theoretically, the incidence and prevalence of OSA should also be increased correspondingly. Nonetheless, the data about epidemiological characteristics of OSA in Chinese population is limited. To what extent OSA potentiates the risk of HTN conferring on the cardiovascular system is unknown and whether this effect is independently associated with SaO_2_ reduction is also less well studied.

We therefore conducted a cross-sectional study to evaluate the association of HTN plus OSA and CVD prevalence in Chinese population, and the extent of this association would also be assessed. Furthermore, whether this association was dependent on mean and lowest SaO_2_ levels would be analyzed too.

## 2 Methods

### 2.1 Studied participants enrollment

Our present research was approved by the clinical research ethic committee of Guangdong General Hospital. Briefly, Guangdong General Hospital is a teaching and tertiary hospital and is also the largest specialty center in South of China. The time of participants’ enrollment was from December of 2013 to September of 2015. All participants were from in-patient cardiovascular ward. Inclusion criteria was the patient agreed to take part in this study and their spouses reported that the patients had ever snored during sleep but had never been diagnosed with OSA previously; and exclusion criteria was the patient disagreed to take in present study, or in a status of acute coronary syndrome, or had been diagnosed as OSA before. Polysomnography would be applied to measure the apnea–hypopnea index (AHI) after informed consent was obtained. Briefly, based on AHI as defined by the total number of apnea (airflow complete blockage for >10 seconds) and hypopnea (> 50% reduction in respiratory airflow accompanying > 3% reduction in SaO_2_ for >10 seconds) per sleep hour,^[16]^ the degree of OSA was classified as mild (AHI 5–14.9) and moderate-severe (AHI ≥ 15), and AHI < 5 was considered without OSA. Both mean and lowest SaO_2_ were detected by pulse oximetry along with polysomnography. All measurements were assessed by using PHILIPS RESPIRONICS Alice PDx.

### 2.2 Demographics, anthropometrics, biochemical data, and medical history collection

Demographics (e.g., age and gender), anthropometries (e.g., weight, height and body mass index, BMI), biochemical data (e.g., lipid profiles), and medical history (e.g., coronary heart disease [CHD] and medications usage) were registered in electronic case report form by 2 working staff and were re-checked by another 2 working staff. Disease condition was confirmed by objective examination plus clinical symptoms. CHD diagnosis was based on coronary angiography, ischemic stroke was based on computer tomography scan plus focal neurologic deficit, and aortic dissection and aneurysm were based on computer tomography scan with contrast infusion. HTN diagnosis was based on either self-report, or treatment with anti-hypertensive drugs, or blood pressure measurement with average value above 140/90 mm Hg. Blood pressure measurement is based on ESH/ESC hypertension guideline recommendation.^[17]^ Before the first reading, patient was required to sit quietly for at least 5 minutes and no smoking was allowed before BP measurement. Appropriate cuff was used, and nondominant arm was selected and placed on a desk in parallel to the level of heart. Three readings with 5 minutes interval were obtained and the value of blood pressure was averaged by the 2 lower readings. Diabetes mellitus diagnosis was based on self-report, or treatment with glucose-lowering drugs, or fasting plasma glucose (FPG) and glycated hemoglobin (HbA1c) values beyond normal range. Dyslipidemia was based on self-report, or treatment with lipid-lowering drugs, or fasting total cholesterol (TC) and low-density lipoprotein cholesterol (LDL-C) levels beyond normal range.

### 2.3 Studied design

Clinical characteristics of participants with and without HTN were initially compared, followed by comparisons of clinical characteristics between participants without OSA and with different degrees of OSA. Thereafter, the additive effects of HTN plus OSA on prevalence of composite CVD were analyzed. Composite CVD comprised CHD, ischemic stroke, aortic dissection, and aneurysm. The association between different subgroups with CVD prevalence was evaluated by logistic regression analysis.

### 2.4 Statistical analysis

Standard descriptive statistics will be applied in the analysis. Continuous variables were described using mean and SD if normal distribution. Otherwise, median and interquartile range (IQR) would be applied. Categorical variables were described by the number and percentages. Variables of AHI and Lp(a) were transformed by Log for statistical analysis. The statistical significance of differences was analyzed using 1-way ANOVA or Mann–Whitney *U* test for continuous variables and the chi-square or Fisher exact test for categorical variables as appropriate. To assess the association between HTN plus OSA and CVD prevalence, logistic regression analysis was applied to calculate odds ratio (OD) and its associated 95% confidence intervals (CI). Statistical analysis would be computed using SPSS 18.0 (SPSS Inc, Chicago, IL). All the statistical tests were 2-sided and considered statistically significant when *P* < 0.05.

## 3 Results

### 3.1 Comparisons between subjects with HTN and subjects without HTN

A total of 1889 recruited subjects were initially divided into no-HTN (40.9%) and HTN (59.1%) groups. As shown in Table 1, hypertensive subjects were older, had higher systolic/diastolic blood pressure (SBP and DBP), HbA1c, and LDL-C levels (*P* < 0.05 for all comparison). Of note, variables closely related to OSA (such as BMI, neck girth, waist–hip ratio, and AHI) were also significantly higher, and mean and lowest SaO_2_ levels were significantly lower in hypertensive subjects (*P* < 0.05 for all comparison), strongly indicating that hypertensive subjects were predisposed to developing OSA. Furthermore, hypertensive subjects also had higher prevalence of diabetes mellitus, ischemic stroke, and aortic dissection (*P* < 0.05 for all comparison). Higher rates of statins usages in hypertensive subjects might correspond to their more co-morbidities including diabetes mellitus and ischemic stroke.

**Table 1:**
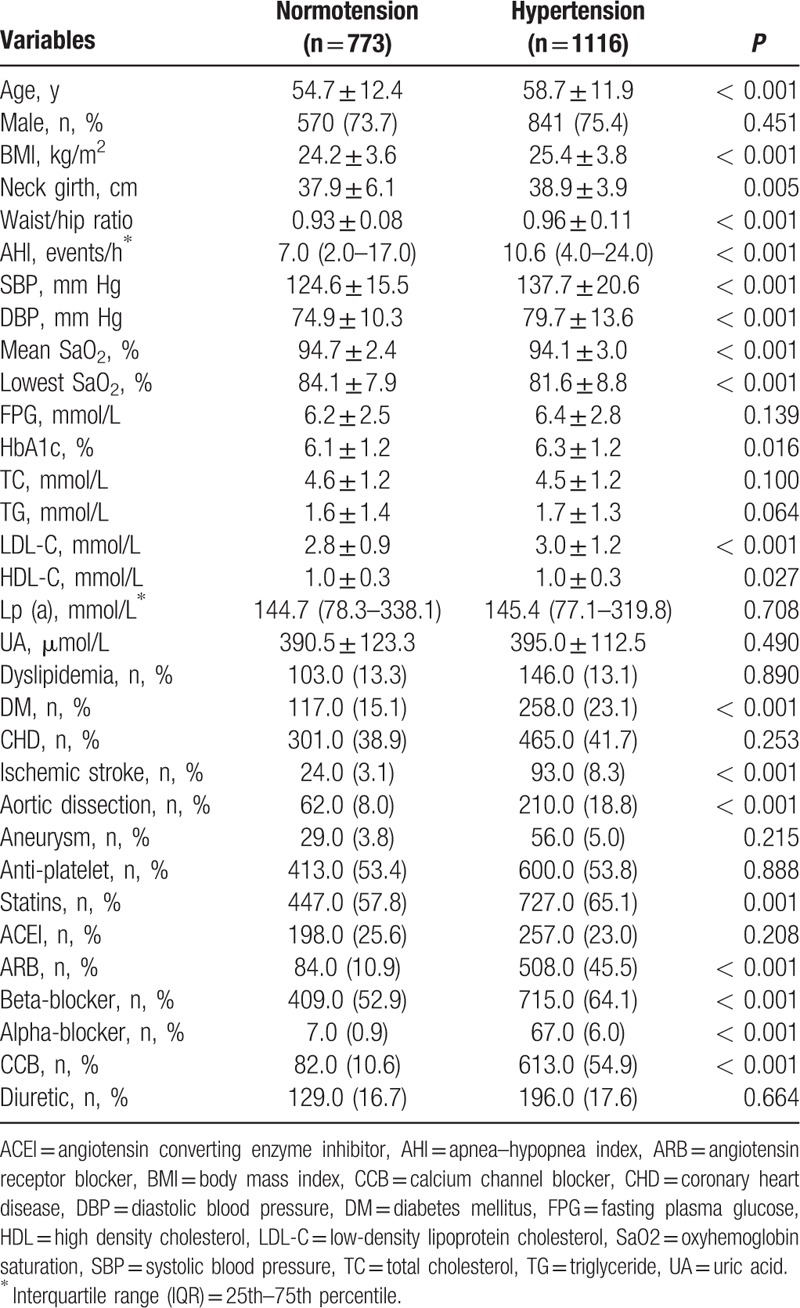
Comparisons between subjects with normotension and with hypertension.

### 3.2 Comparisons between subjects with different degrees of OSA

In order to evaluate the differences in clinical characteristics (especially those related to CVD) between subjects with different degree of OSA, 1889 recruited subjects were separated into 3 groups based on AHI as mentioned above. As expected, variables closely related to OSA (such as BMI, neck girth, waist–hip ratio, and AHI) were significantly higher, whereas mean and lowest SaO_2_ levels were significantly lower in moderate-severe-OSA subjects (*P* < 0.05 for all comparison) as shown in Table 2. In addition, compared to subjects without OSA or subjects with mild-OSA, those with moderate-severe-OSA were older, predominantly male, had considerably higher FPG, HbA1c, triglyceride (TG), and uric acid (UA) levels. Furthermore, prevalence of co-morbidities including HTN, diabetes mellitus, and CHD were also significantly higher (*P* < 0.05 for all comparison).

**Table 2:**
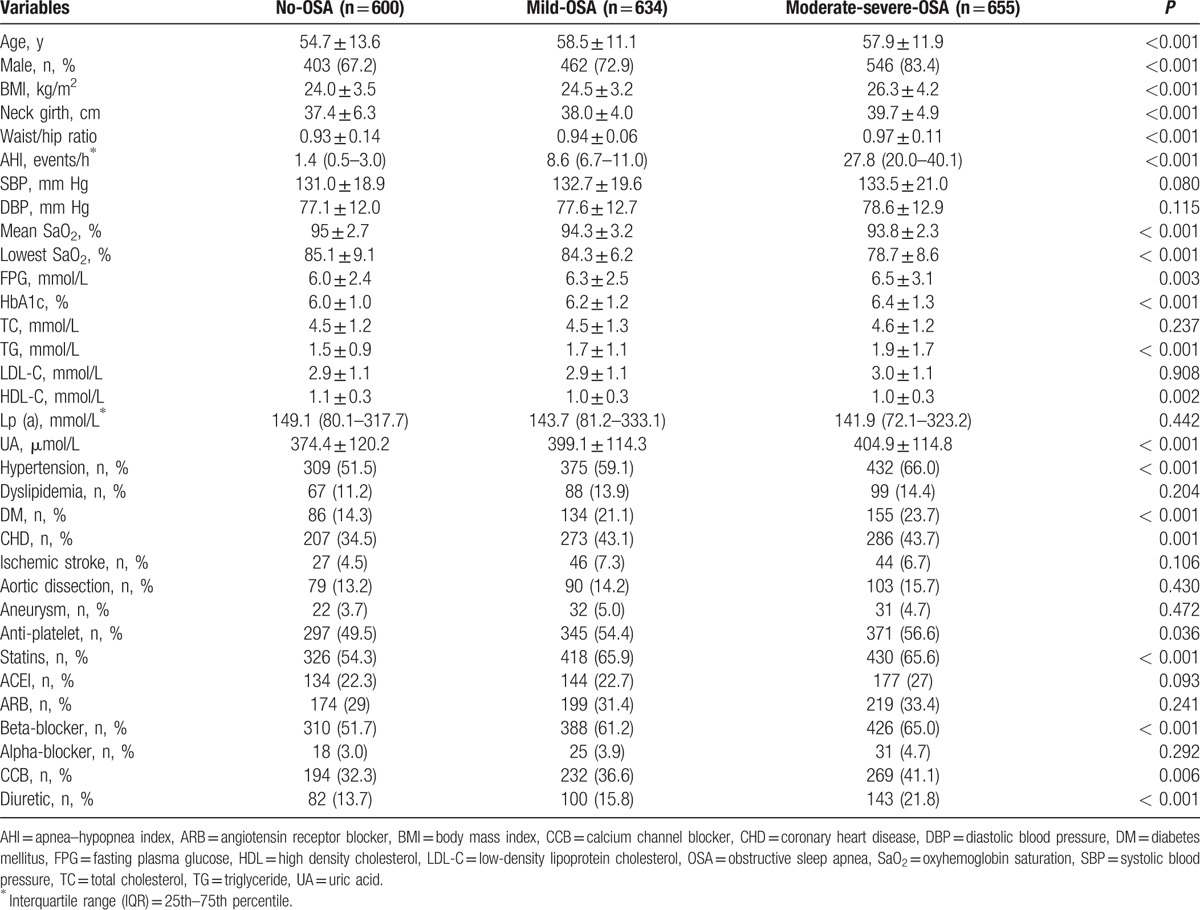
Comparisons between subjects with different degrees of OSA.

### 3.3 Associations of OSA plus HTN and CVD prevalence

Based on different stratifications, participants were classified into 6 subgroups as follows: no-HTN plus no-OSA (n = 291), no-HTN plus mild-OSA (n = 259), no-HTN plus moderate-severe-OSA (n = 223), HTN plus no-OSA (n = 309), HTN plus mild-OSA (n = 375), and HTN plus moderate-severe-OSA (n = 432). Logistic regression analysis was applied to evaluate the association of OSA plus HTN and CVD prevalence. Subjects without OSA and HTN (no-OSA plus no-HTN) were defined as the reference group. In the unadjusted model, compared to the reference group, subjects with either OSA or HTN were at higher CVD prevalence, with strongest association in subjects with HTN plus moderate-severe-OSA with OR of 2.638 (95% CI: 1.942–3.583). In normotensive subjects, after adjusted for age, gender, BMI, neck girth, waist–hip ratio, FPG and LDL-C (model 1), the association of OSA (regardless of severity) and CVD prevalence were attenuated to nonsignificant. In hypertensive subjects, nevertheless, the association of OSA and CVD prevalence remained significant although were attenuated as presented in Table 3. After further adjusted for mean and lowest SaO_2_ (model 2), the associations remained significant in HTN plus no-OSA (OR: 1.808, 95% CI: 1.207–2.707), HTN plus mild-OSA (OR: 2.003, 95% CI: 1.346–2.980), and HTN plus moderate-severe OSA (OR: 1.834, 95% CI: 1.214–2.770) groups.

**Table 3:**
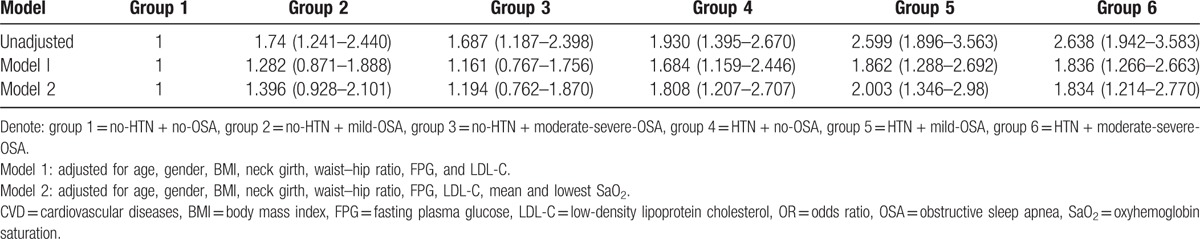
Crude and adjusted OR for prevalent CVD across different groups.

## 4 Discussion

The principal findings of our current study include 2 aspects. First, our current study is in concert with previous findings that in Chinese populations HTN plus OSA is associated with higher prevalence of CVD, and second, this association is independent of mean and lowest SaO_2_ levels.

Both HTN and OSA have been deemed as major risk factors of CVD, and linear relationship between both blood pressure and AHI with CVD prevalence has also been demonstrated in previous epidemiological studies.^[18,19]^ Consistent with previous findings, data from our current study also supported the concept that the OSA degree is positively associated with CVD risk. The following aspects might explain these findings. On the one hand, reduced SaO_2_ might play a central role. As is well known that nocturnal SaO_2_ reduction is a sensitive marker indicating the severity of OSA-associated intermittent hypoxemia, and pathological alterations such as systemic inflammation, oxidative stress, and endothelial dysfunction ensued are associated with CVD development.^[20]^ For example, Ryan and colleagues reported that selective activation of inflammatory pathway in response to intermittent hypoxemia was associated with high prevalence of CVD in OSA patients.^[7]^ And oxidative stress induced by intermittent hypoxemia is capable of aggregating the deleterious effects incurred from OSA risk factors such as obesity and HTN.^[8]^ Dose–effect relationship between SaO_2_ or AHI events and CVD risk additionally supported the hypothesis that apnea/hypopnea-related SaO_2_ reduction was associated with prevalent CVD. For example, Punjabi et al^[21]^ reported that hypopnea with a de-saturation of at least 4% was independently associated with CVD and no association was observed between CVD and hypopnea-associated modest de-saturation. Another epidemiological study conducted by Sampol et al^[22]^ revealed that there was an association of OSA and thoracic aortic dissection, and patients with aortic dissection were suffering an average of 28 events per sleep hour which was significantly higher than their controls (11.1 events per sleep hour). With respect to our finding, compared to no-OSA and mild-OSA groups, both mean and lowest SaO_2_ levels were significantly lower in the moderate-severe-OSA group, which might be at least partially explain the higher CHD prevalence. On the other hand, OSA-related co-morbidities such as increased BMI and central adiposity, impaired fasting glucose, diabetes mellitus, and dyslipidemia might also aggregate the adverse effects of OSA exerts on the cardiovascular system. Information from this part offered preliminary data regarding the clinical characteristics of OSA in Chinese populations (both hypertensive and normotensive), which may be helpful for future prospective studies in designing screened algorithm.

We did not stratify studied subjects into different blood pressure value groups. Rather, we merely divided subjects into no-HTN and HTN groups since only blood pressure value at admission was taken into analysis. Notwithstanding that, a substantial number of epidemiological studies could justify our findings that hypertensive subjects were indeed at greater CVD risk than their normotensive counterparts. Furthermore, as expected, hypertensive subjects were predisposed to developing OSA as featured by a cluster of OSA risk factors including higher BMI, neck girth, and waist–hip ratio. Information from this part provided evidence regarding increased risk of OSA development in hypertensive subjects, which may be helpful to raise concerns about the benefits of OSA screening in hypertensive patients especially those with OSA trait.

The association of HTN plus OSA and prevalent CVD was further evaluated. Coronary heart disease, ischemic stroke, aortic dissection, and aneurysm were defined as composite CVD due to high prevalence as well as great burdens of these diseases together imposing on individual patients and the whole society. In addition, from statistical aspect, combined individual outcome together could help improve power to detect modest between-group difference when subjects were divided into several subgroups causing relatively small number of event in each subgroup. In logistic regression analysis, the crude OR value indicated that the association of HTN plus moderate-severe OSA and CVD prevalence was the most strongest. Theoretically, the concept that the more severe OSA (as indexed by higher AHI event) the higher CVD risk might justify this observation. Nevertheless, after adjusted for potential confounding factors, these associations were attenuated with HTN plus moderate-severe OSA was diminished most prominently. From statistical aspect, this finding might be due to the interactive effect of obesity (indexed as BMI) and central adiposity (indexed as waist–hip ratio) together exert on OSA and HTN.^[23,24]^ Indeed, significantly higher BMI and waist–hip ratio in subjects with moderate-severe-OSA might also support this speculation. Interestingly and importantly, the associations of prevalent CVD with HTN plus no-OSA and HTN plus mild-OSA groups were enhanced but not in HTN plus moderate-severe-OSA group after additional adjusted for mean and lowest SaO_2_ as presented in Table 3, indicating that the association of OSA plus HTN and CVD prevalence were independent of OSA severity (as indexed by the mean and lowest SaO_2_ levels). Nevertheless, this unexpected finding might also be attributed to small numbers of studied subjects which leaded to unstable estimates. Over-adjustment might also be a reasonable explanation since all included confounding factors were closely correlated with each other. Nevertheless, these factors were also independently associated with CVD which might not be appropriate to exclude.

Several limitations of current study merited address. First of all, the nature of observational design would not allow us to draw causal relationship between HTN plus OSA and CVD prevalence. Second, only blood pressure value at admission was taken into analysis which could not sufficiently reflect the synergistic effect of HTN (especially nocturnal HTN) and OSA on CVD prevalence. In addition, the white-coat effect was also could not be excluded. Nonetheless, since standardized and similar BP measurement method was applied to all participants and thus potential bias between study groups could be avoided. Last but not the least, only mean and lowest SaO_2_ levels were included in final analysis and the duration of de-saturation was not available which could not allow us to evaluate the comprehensive effects of oxyhemoglobin reduction on CVD prevalence.

## 5 Conclusion

Results from our current study show that there may be synergistic adverse effects of OSA and HTN on the cardiovascular system which is independent of mean and lowest SaO_2_ levels.
